# Involvement of Resveratrol against Brain Cancer: A Combination Strategy with a Pharmaceutical Approach

**DOI:** 10.3390/molecules27144663

**Published:** 2022-07-21

**Authors:** Chenmala Karthika, Agnieszka Najda, Joanna Klepacka, Mehrukh Zehravi, Rokeya Akter, Muhammad Furqan Akhtar, Ammara Saleem, Majed Al-Shaeri, Banani Mondal, Ghulam Md. Ashraf, Priti Tagde, Sarker Ramproshad, Zubair Ahmad, Farhat S. Khan, Md. Habibur Rahman

**Affiliations:** 1 Department of Pharmaceutics, JSS College of Pharmacy, JSS Academy of Higher Education & Research, Ooty 643001, India; karthika1994haridas@gmail.com; 2 Department of Vegetable and Herbal Crops, University of Life Science in Lublin, Doświadczalna Street 51A, 20280 Lublin, Poland; 3 Department of Commodity Science and Food Analysis, Faculty of Food Science, University of Warmia and Mazury in Olsztyn, Oczapowskiego 2, 10719 Olsztyn, Poland; klepak@uwm.edu.pl; 4 Department of Clinical Pharmacy Girls Section, Prince Sattam Bin Abdul Aziz University, Alkharj 11942, Saudi Arabia; mahrukh.zehravi@hotmail.com; 5 Department of Global Medical Science, Wonju College of Medicine, Yonsei University, Wonju 26426, Korea; rokeyahabib94@gmail.com; 6 Riphah Institute of Pharmaceutical Sciences, Lahore Campus, Riphah International University, Lahore 54950, Pakistan; furqan.pharmacist@gmail.com; 7 Department of Pharmacology, Faculty of Pharmaceutical Sciences, Government College University Faisalabad, Faisalabad 38000, Pakistan; ammarasaleem@gcuf.edu.pk; 8 Department of Biological Sciences, Faculty of Science, King Abdulaziz University, Jeddah 21589, Saudi Arabia; malshaere@kau.edu.sa; 9 Department of Pharmacy, Ranada Prasad Shaha University, Narayanganj 1400, Bangladesh; banani091110@gmail.com (B.M.); ramproshad131135@gmail.com (S.R.); 10 Pre-Clinical Research Unit, King Fahd Medical Research Center, King Abdulaziz University, Jeddah 21589, Saudi Arabia; ashraf.gm@gmail.com; 11 Amity Institute of Pharmacy, Amity University, Noida 201301, India; tagde_priti@rediffmail.com; 12 Unit of Bee Research and Honey Production, Faculty of Science, King Khalid University, P.O. Box 9004, Abha 61413, Saudi Arabia; dzubair@gmail.com; 13 Biology Department, College of Arts and Sciences, Dehran Al-Junub, King Khalid University, P.O. Box 9004, Abha 61413, Saudi Arabia; farhatamu@gmail.com

**Keywords:** brain tumor, blood–brain barrier, P-glycoprotein, antioxidants, resveratrol, nasal delivery

## Abstract

A brain tumor (BT) is a condition in which there is growth or uncontrolled development of the brain cells, which usually goes unrecognized or is diagnosed at the later stages. Since the mechanism behind BT is not clear, and the various physiological conditions are difficult to diagnose, the success rate of BT is not very high. This is the central issue faced during drug development and clinical trials with almost all types of neurodegenerative disorders. In the first part of this review, we focus on the concept of brain tumors, their barriers, and the types of delivery possible to target the brain cells. Although various treatment methods are available, they all have side effects or toxic effects. Hence, in the second part, a correlation was made between the use of resveratrol, a potent antioxidant, and its advantages for brain diseases. The relationship between brain disease and the blood–brain barrier, multi-drug resistance, and the use of nanomedicine for treating brain disorders is also mentioned. In short, a hypothetical concept is given with a background investigation into the use of combination therapy with resveratrol as an active ingredient, the possible drug delivery, and its formulation-based approach.

## 1. A Basic Outline on Resveratrol

Resveratrol (RES) is a polyphenol extracted from mulberries (*Morus species*), grapes (*Vitis vinifera*), and peanuts (*Arachis hypogaea*). It is a phytoalexin that spermatophytic plants make in reaction to stress, damage, or UV radiation, as well as fungus (e.g., Botrytis cinerea) and/or another pathogen [1]. Several biotic and abiotic factors have been investigated in the context of induced RES production in a variety of plants. Plants synthesize RES via the phenylalanine route [2]. Resveratrol 3-O-beta-glycosyltransferases convert the end product to trans form, which can subsequently isomerize to cis form or trans and cis-priced. Furthermore, the stilbene route is a branch of the phenylpropanoid system, which is an extension of the flavonoid pathway [3]. Michio Takaoka isolated RES from the root of *Veratrum grandiflorum O*. Loes in 1939. In 1963, RES was determined to be a chemical component of *Polygonum cuspidatum* (Ko-jo-kon). The first known detection technique for trans-resveratrol was published in 1976. Renaud and de Lorgeril first published the “French paradox” in 1992, after which RES faded into oblivion [4]. The French paradox is based on epidemiological evidence from French people who had a high-fat, high-cholesterol diet while having a low incidence of coronary heart disease. In reality, as compared to the United Kingdom, the United States, or Sweden, France continues to have a low incidence and mortality rate from coronary heart disease. The concentration of RES in various wines was examined the same year [5]. A 2001 study discovered a link between moderate-to-low wine consumption and a decreased risk of mortality from cardiovascular and cerebrovascular disorders. Following these discoveries, the French paradox sparked widespread interest, with hundreds of research projects conducted on various parts of it [6]. RES has been proven to have antioxidant, anti-inflammatory, cardioprotective, and analgesic properties, as well as a function in diabetes and obesity. Because of its growing importance in neurological illnesses such as Parkinson’s, Alzheimer’s, and other neurodegenerative disorders, as well as BT, RES has received a lot of attention [7]. RES has also been shown to have anticancer characteristics in a variety of different malignancies, including prostate cancer, liver cancer, breast cancer, lung cancer, skin cancer, and colorectal cancer [8].

## 2. A Background Search on Brain Cancer

**Figure 1:**
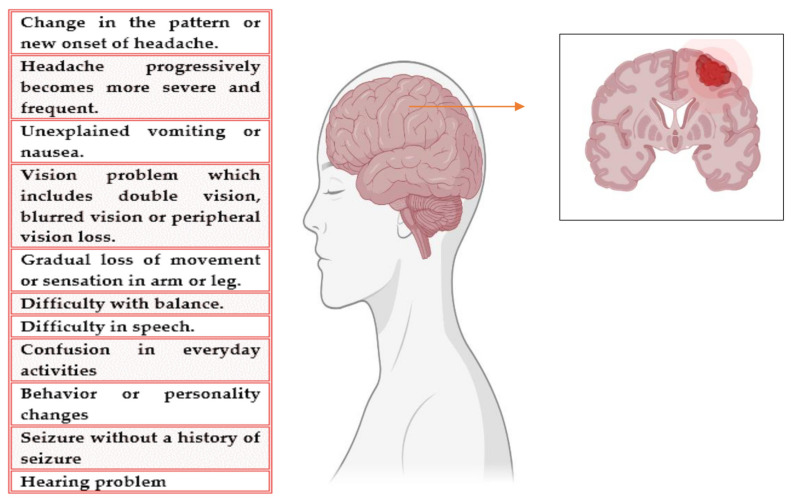
Symptoms of BT.

A brain tumor (BT) is an unconditional development of cells in the brain [9]. The symptoms of BT are difficult to diagnose, and the symptoms worsen during the progression of this disease. BT can originate in the brain (known as primary BT) or spread from other parts of the body (known as secondary or metastatic BT). The growth and location in the brain determine the response of cancer to nervous functioning [10]. The symptoms and mitigation options are also contingent on the size and location. The signs and symptoms of BT are shown in Figure 1. The condition or state in which a tumor cannot be detected in our body is called remission. Another problem that causes treatment failure is the recurrence of cancer after treatment [11]. The risk of reoccurrence can be in the original location or other parts of the body. If the tumor spreads, that is, when metastasis occurs, then the success rate of the treatment is reduced to 15% [12]. It is not always possible to recover from BT. If the tumor cannot be controlled or cured, then the disease may be called terminal or advanced. When diagnosed with advanced BT, the expected life span is calculated to be less than 6 months [13].

Primary BTs generally initiate in the brain or the tissues closer to the brain, such as the pituitary gland, cranial nerves, pineal gland, or meninges-brain covering membrane. The origin of BT is generally a mutation in the DNA, which results in the uncontrollable division of the brain cells and the death of the healthy cells [14]. In adults, primary BT is more prevalent than secondary BT.

**Figure 2:**
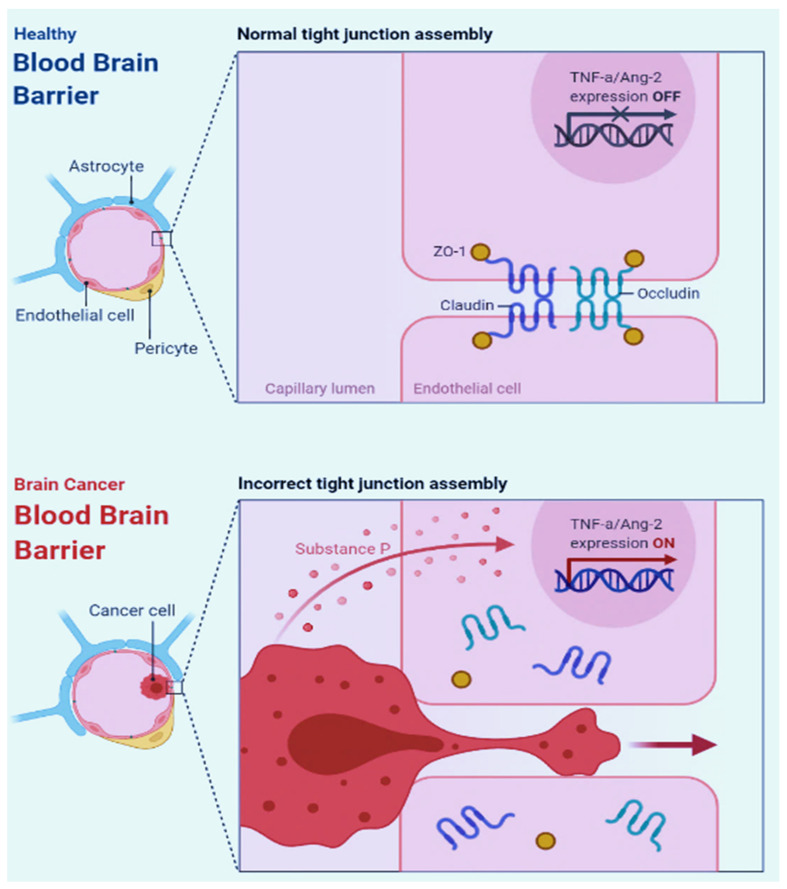
The difference between healthy BBB (BBB) and BT BBB.

Cancer growth can reduce blood flow even in perineural regions by compressing blood vessels due to the limited space in the brain. Furthermore, the neurovascular unit develops differently in the cancer core compared to the cancer periphery and neuroparenchyma, the latter of which has an intact blood–brain barrier (BBB) as BT lesions grow [15]. In Figure 2, the difference between the healthy BBB and the BT BBB is shown.

The cancer vasculature becomes increasingly heterogeneous as the primary BT progresses and brain metastasis develops. Neuronal viability and vascular function are directly affected by local and distal changes caused by an expanding neoplastic lesion [16]. Because the vasculature changes during cancer growth, existing vessels must be co-opted, and/or new ones must be created through angiogenesis to meet the nutritional needs of proliferating cancer cells. Postnatal vasculogenesis, vascular mimicry, intussusception, and transdifferentiation are some of the other mechanisms by which cancers increase blood vessel supply [17]. Cancer progression is accompanied by vascular dysfunction and an acidic microenvironment, which are fueled in part by hypoxia-inducible factor 1 (HIF1) transcriptional programs. Studies reported that when vascular endothelial growth factor (VEGF) signaling is blocked in mice, BTs are lopped into their immature and leaky vessels, and the remaining vasculature is actively remodeled to look like normal vasculature [18].

Surgery, chemotherapy, radiotherapy, and targeted therapy are some of the treatment options for BT. The option used for low-grade cancer is mostly surgical removal of the tumor [19]. Medications are used as a treatment option to destroy cancer cells, categorized as chemotherapy and targeted therapy. A chemotherapeutic regimen is used to inhibit cancer cell division and the growth of the cancer cells, which can destroy the cancer cells and reduce the symptoms. Sometimes, chemotherapy is suggested after radiotherapy or surgery. Drugs that can bypass the BBB are commonly used as BT drugs, such as canmustine, temozolomide, and combinations of vincristine, lomustine, and procarbazine [20]. If the growth of the cancer cells is not retarded, then other treatment options are taken into consideration. In addition to standard chemotherapy, an alternative is targeted therapy, such as anti-angiogenesis therapy or therapy that focuses on genetic changes.

The chemical-based treatment strategy has various side effects and toxic effects, which include hair loss, nausea, vomiting, fatigue, diarrhea, loss of appetite, etc. [21]. Some drugs may even cause hair loss, kidney damage, and increase the risk of infection [22]. Current research focusing on natural remedies for mitigating BT lags behind, partially because there is no clear-cut idea about the BT mechanism itself. However, herbal and natural remedies may help to reduce the risk of BT, ease some of its symptoms, and even inhibit cell growth [23]. Herbal remedies are generally recommended as safe, even under long use and in larger doses. Hence, the combination of a drug or a natural compound and a chemotherapeutic drug could be a possible effective therapeutic modality for the mitigation of BT.

In this paper, a hypothetical concept is provided with a background investigation of BT and the combination approach with the use of resveratrol. The drug delivery design is made with the possible route of administration in mind. This article provides a background for researchers and the concept of drug formulation using resveratrol for BT.

## 3. Methodology

**Figure 3:**
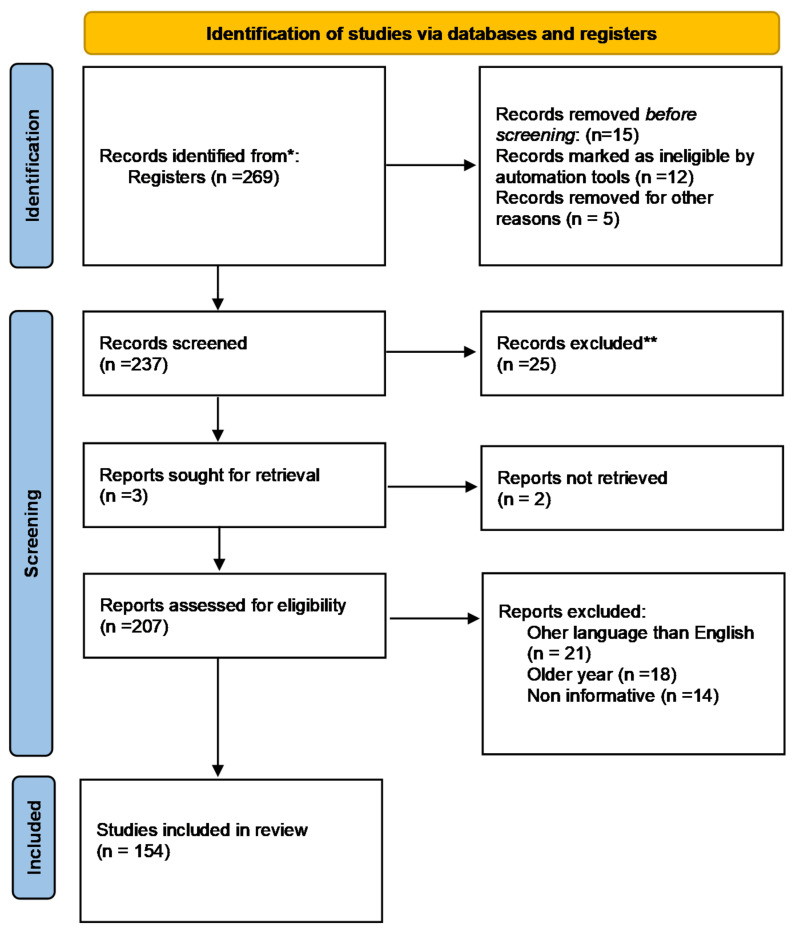
PRISMA flowchart describing the reference selection procedure. * PubMed, Web of Science, Cochrane Library, Google scholar, etc. ** repeated, irrelevant or duplicate information.

The authors conducted extensive research of the published literature on this topic, choosing scientific papers that address resveratrol implications in BTs, pathways of action, route of administration, cellular pathways, and apoptosis while emphasizing the most relevant and interesting aspects. The publishing intervals for the selected papers were infinite, and all publications produced this year were treated identically (until the submission date of the current study). To obtain precise and full information, the most well-known medical and biological databases (PubMed, Web of Science, Cochrane Library, Google scholar, and so on) were explored. To indicate the scope of the effort, in May 2022, a search on PubMed for the terms “resveratrol” and “cancer” yielded 4229 results, and resveratrol and BT generated 167 results. Figure 3 (a PRISMA flow chart) summarises the criteria used to choose the right bibliography and shows the entire process clearly using Page et al.’s recommendations [24]. The most crucial keywords (resveratrol, natural compound, BT prevention, molecular signals, cell signaling, combination therapy, apoptosis, and others) and the Medical Subject Heading (MeSH) phrases were used to search for the most relevant published data. The articles judged eligible were picked initially based on their title, keywords, and abstract; subsequently, the content analysis was decisive, aided by filtering tools (i.e., Clinical Queries). The source was utilized as a reference, and the most useful and relevant results and data were extracted.

## 4. Correlation between Oxidative Stress, Antioxidants, and BT

According to various studies, resveratrol is an antioxidant, anti-inflammatory, and anticancer agent [25]. Because mitochondria play a role in both healthy and diseased states, researchers are also looking into the effect of RES on mitochondrial function and dynamics. RES, among other things, affects mitochondrial function, redox status, dynamics, and synthesis [26]. Mitochondrial dysfunction has been identified as a critical component of neurodegeneration in neuronal and glial cells, and dietary modulation of mitochondria-related variables is an appealing technique for preventing or treating neurodegenerative illnesses [27].

### Oxidative Stress

Oxidative stress is believed to act on cancer formation. Oxygen is required for respiration and energy processes, and a balance between antioxidant stress and the production of reactive oxygen species (ROS) is considered to be necessary for healthy aerobes. Oxygen-free radicals play a critical role in various pathological conditions such as neurological disorders, cardiovascular disease, cancers, and aging [28]. Because the brain has a higher rate of anaerobic respiration than other organs, the oxidative damage framework is particularly concentrated in BTs. Moreover, the larger amount of DNA damage formed by ionizing radiation is the only significant effect that produces damage for BTs, which is assumed to be produced by oxidative reactions [29]. Despite substantial scientific proof, the immune system plays a major role in BT etiology. On the other hand, it is also important to note that ROS acts as a barrier against infection and facilitates the immune reaction. Several enzymes act to mitigate or prevent the damage caused by ROS, including catalase (CAT), nitric oxide synthase (NOS), superoxide dismutase (SOD), glutathione peroxidase (GPX), and paraoxonase (PON) [30].

ROS is an entirely unnecessary byproduct of metabolites and plays an important role in many biological mechanisms [31]. However, it must be accurately captured. A wide range of plant species rich in phytochemical antioxidants has been associated with anti-cancer characteristics in the past. There are legitimate researches and vocational interests in determining powerful anti-cancer molecules obtained from natural sources, such as secondary plant metabolites, for the mitigation and prevention of various disorders [32]. Food-derived polyphenol antioxidants have anti-cancer properties by lowering ROS-induced oxidative damage via scavenging or boosting endogenous antioxidants. Natural antioxidants have the potential to affect signal transduction pathways by activating or inhibiting a variety of redox-responsive transcription factors, indicating that they are used as chemopreventive agents [33]. They also might play a role in carcinogenesis initiation, promotion, and progression stages, as well as preventing cancer growth. Antioxidant molecules prevent, reduce, or eradicate oxidative stress at a binding site. The brain is extremely vulnerable to ROS destruction due to its low antioxidant levels [34]. The brain requires adequate manufacturing of antioxidants to recover from ROS injury, and it certainly appears that cooperating with ROS cellular functions can provide important neuroprotection [35]. Furthermore, the prevention and mitigation moiety must consider various biochemical pathways involved in cancer while mitigating negative impacts and toxic responses in normal tissue equally [3]. On the other hand, an advantageous task in enhancing the therapeutic benefit of chemotherapeutics and reducing adverse properties should also be addressed.

Flavonoids can be found in a variety of foods, including fruits, wine, cereals, vegetables, tea, and fruit juices. Due to excessive metabolism, flavonoids lose a significant amount of their potent antioxidant properties during absorption. Many flavonoids-glycosides and aglycones found in plant-based foods are heavily conjugated and metabolized during absorption [36]. It is critical to determine whether flavonoids and their metabolic derivatives can enter the central nervous system and act as neuromodulators. Flavonoids must cross the blood–brain barrier before they can enter the BBB [37]. Preventing xenobiotics from entering the brain and preserving its microenvironment are two of its functions. The lipophilicity of the flavonoid compound appears to affect its penetration. Interactions with specific efflux transporters expressed in the BBB, in addition to lipophilicity, may influence flavonoids’ ability to enter the brain [38]. P-glycoprotein is a transporter involved in drug absorption and brain uptake. P-gp coexists with the BBB for its protective function [39]. It has also been demonstrated that flavonoid localization improves cognitive performance, implying that these components have neuroprotective properties. Even though flavonoids and their metabolites appear to reach the brain, their levels in the body are significantly lower (high nM, low mM). According to research, flavonoids’ cellular responses may be mediated by the mitogen-activated protein (MAP) kinase signaling pathway and the phosphoinositide 3-kinase (PI3 kinase/Akt) signaling cascade [40].

## 5. RES—A General Concept and a Basic Outline

RES is a plant-derived polyphenol found in berries, grapes, nuts, and red wine [1]. RES crosses the BBB, which may have an impact on the nervous system [41]. It also affects the enzyme isocitrate dehydrogenase and, more importantly, resistance to standard mitigation through a variety of mechanisms, including O6-methylguanine methyltransferase. For example, RES was shown to inhibit resistance to standard alkylating agents such as temozolomide by inhibiting O6-methylguanine methyltransferase activity [42].

RES is a low-toxicity molecule that targets specific potential biological signaling pathways and affects several carcinogenesis-related genes [43]. The important antiproliferative capability of RES was already indicated in a diverse array of cancer forms when administrated alone or in addition to various anticancer agents and targeted therapies. Its ability to prevent BT includes the inhibition of oxidative stress and inflammation and also the regulation of cell proliferation through the signaling of apoptosis pathways. It may influence the activity of cancer cells by affecting various signaling mechanisms such as PI3K/AKT/mTOR, nuclear factor-kB (NF-kB), p53, Wnt, or even signal transducer and activator of transcription-3 (STAT3).

### 5.1. Resveratrol and Its Pharmacological Action in the General Aspect

#### 5.1.1. Resveratrol’s Pharmacological Influence on Immunity

The first research of RES on immunomodulatory effects revealed that it suppressed interleukin-2-induced (IL-2) and spleen cell proliferation. It has a greater impact on lymphocyte and macrophage production of IL-2, IFN-β, and TNF/IL-12 [44]. RES is either directly or indirectly involved in the establishment and modulation of innate and adaptive immunity [45]. The immune system is affected by RES in a dose-dependent manner. At low dosages, it activates the immune system for an immunological response, while at higher levels, it suppresses the immune system [46]. Another research discovered that it promoted chicken growth by increasing immune responses and decreasing immunocyte mortality. RES inhibited the Toll/IL-1 receptor, reducing the activity of the respiratory syncytial virus [47]. RES suppressed enterovirus replication and reduced the virus’s generation of IL-6 and TNF-α [48]. RES increased CD4+ cell proliferation, splenic lymphocyte proliferation, and peritoneal macrophage activity in mice [49]. Intravenous RES therapy decreases ischemia-induced inflammation and oxidants produced by HX/XO, but not leukotriene B4 [50].

#### 5.1.2. Resveratrol’s Effect on Cancer

**Figure 4:**
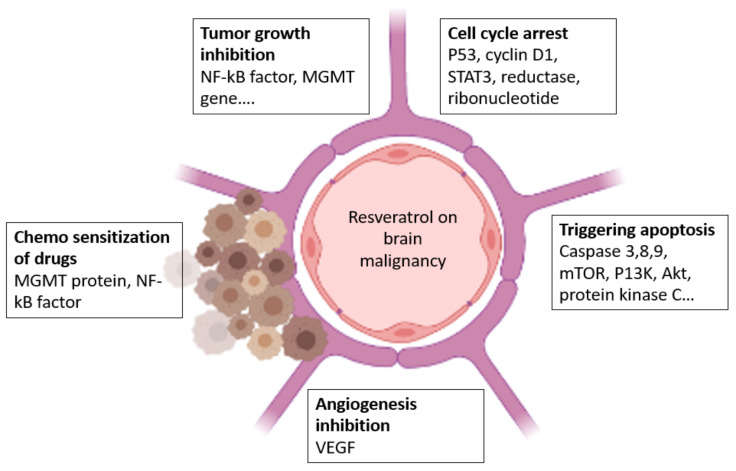
Effect of resveratrol on BT.

Jang et al. discovered that RES could suppress carcinogenesis in mouse skin cancer models, and further research has confirmed this conclusion [51]. Later, it was shown that RES inhibited cancer cell growth in the ovary, stomach, intestine, prostate, colon, liver, pancreas, brain, and thyroid. In HL-60 leukemic cells, RES decreased free radical production caused by 12-O-tetradecanoylphorbol-13-acetate [52]. By scavenging OH and superoxide generated by cells, as well as lipid peroxidation occurring inside cell membranes, RES protects against DNA damage caused by ROS production. The antimutagenicity of N-methyl-N-nitro-N-nitrosoguanidine in *S. Typhimurium strain* TA100 was shown using RES. A recent study in colorectal cancer has revealed that RES is a multifunctional factor with anti-inflammatory and anticancer properties [53]. They have also been shown to block the NF-kB signaling pathway. RES has also been demonstrated to inhibit TNF-α stimulated colorectal cell invasion and viability. RES also suppressed the activation of NF-kB and carcinogenic gene products in colorectal cells, as well as other signaling molecules such as vimentin, slug, and E-cadherin [54]. The concept of the action of resveratrol on BT cells is given in Figure 4.

#### 5.1.3. Toxicity Effects of Resveratrol

Several researchers have found that RES has harmful impacts. After 24 h, no adverse effects were seen in 15 healthy volunteers who received a single high dose (500 mg) of RES while fasting. Long-term therapy with RES at a dosage of 2.5 g per day, however, produced vomiting, diarrhea, and nausea in healthy patients [55]. Surprisingly, long-term (up to one year) intake of grape RES at levels as high as 16 mg exhibited no discernible negative effects. After a dose of 5 g RES in the form of SRT501 (developed by Sirtris, a GSK company) was administered in two cycles during multiple myeloma, renal toxicity was observed in healthy controls, type 2 diabetics, and patients with lactic acidosis, mitochondrial encephalomyopathy, and stroke-like episodes (MELAS) syndrome. The researchers concluded that the appropriate amount of RES is required to treat a range of disorders [7].

## 6. RES Brain Delivery

RES use has been linked to several health benefits, including anti-inflammatory, anti-carcinogenic, and brain inhibitory responses [56]. The neuroprotective responses of RES in neurological diseases like Alzheimer’s (AD) [57] and Parkinson’s (PD) [58] are signified by the establishment of neurons that are protected from oxidative damage and toxicity, as well as the prevention of apoptotic neuronal death [59]. In BTs, RES induces cell apoptosis while inhibiting angiogenesis and cancer invasion. Despite its enormous potential as a therapeutic agent for a wide range of diseases, RES has some barriers [4]. It is chemically unbalanced and is degraded by isomerization when unprotected from high temperatures, pH changes, UV light, low water solubility, or specific enzymes [60]. As a result, RES has limited biological, low bioavailability, and pharmacological benefits. To get around these restrictions, RES can be carried via nanocarriers [2]. This subfield of nanomedicine studies how the use of nanoscale materials affects pharmacokinetics, drug administration, and pharmacodynamics [61]. The use of nanotechnology in the mitigation and prevention of neurological diseases also conceals the physicochemical assets of therapeutic drugs to enhance their half-life and cross the BBB. This is essentially achieved by encapsulating the drug in a nanoparticle (NP) composed of an array of substances [62]. There is a rising trend to encapsulate and deliver RES to the brain. RES encapsulated liposomes, lipid nanoparticles, and polymeric nanoparticles are being used to encapsulate RES. Furthermore, the majority of these nanocarriers have been obtained by targeting molecules that can recognize brain regions [3]. In both in vitro and in vivo experimental models, RES has been shown to regulate redox biology, mitochondrial function, and dynamics [63]. RES also mitigates the mitochondrial dysfunction caused by oxidative stress. RES regulates the release of mitochondrial antioxidant enzymes, limiting reactive species stimulation by these organelles [64]. RES also promotes mitochondrial biogenesis, which enhances mitochondrial bioenergetics in mammalian cells. The researchers discussed the responses of RES to brain mitochondria. Brain cells (both neuronal and glial) are vulnerable to mitochondrial dysfunction due to their high demand for ATP [65]. Moreover, brain cells produce a lot of oxygen, which causes the mitochondria to generate a lot of reactive species. As a result, strategies focusing on mitochondrial function restoration in these types of cells are of therapeutic interest in neurodegenerative diseases requiring mitochondrial impairment and enhancing reactive species capacity, resulting in neuroinflammation and cell death [66]. The mechanism by which RES protects mitochondrial function and dynamics is unknown, and more research is needed to determine how RES affects mitochondrial-related factors. Moreover, it is critical since RES, depending on the dosage, can cause cytotoxicity [1].

## 7. Barriers

As a result of hypoxia, an increase in cancer cell invasiveness is an unintended side response of anti-angiogenic therapy [67]. High-dose anti-angiogenic may reduce blood-BT barrier (BTB) permeability, jeopardizing the long-term delivery of other therapeutics. It is difficult to strike the right balance between these vascular phenotypes while treating primary BTs and brain metastases with anti-angiogenic therapies [68]. The BBB is a threat to medical interventions. With research into the structure and function of the BBB/BTB, drugs can now be delivered to BTs, including those that have invaded the perineural regions [69].

Small hydrophobic molecules with a molecular weight of less than 500 Da (less than 1 nm) diffuse transcellularly into the neuroparenchyma when the BBB is intact [70]. Various researchers stated that many circulating pharmacological compounds bind to multidrug-resistant ATP-binding cassette (ABC) transporters [71]. As a result, ABC transporters help to reduce the rate at which potential drugs cross the BBB while also helping to improve the BTB’s barrier properties [72]. To circumvent or hijack the BBB’s cellular and molecular barriers, a variety of strategies are being developed or optimized. Improved drug delivery across the BBB/BTB can be accomplished through the use of both invasive and non-invasive techniques. Even though invasive approaches necessitate direct access to the disease site, they are still being refined and optimized, with promising preclinical results [73].

## 8. Combination, Synergistic Response, and the Response to Conventional Therapy

A combination strategy is proposed to produce a synergistic response. In this concept, two herbal ingredients or an herbal and a synthetic agent could be combined either by physical or chemical conjugation [74]. The combination of the herbal and synthetic agents is found to be best for the mitigation of BT as it could reduce the toxic response of the synthetic agent, which is achieved by reducing the dose [75].

The synergistic activity of the combination can be confirmed by using in vitro cell line studies. In addition, analytical methods are being used to confirm the compatibility of the drugs [71]. Focus on the combination strategy when used with a synthetic chemotherapeutic agent can help in dose reduction and, hence, the toxic response can also be reduced. The use of herbal remedies mainly acts as a P-gp inhibitor; hence the efflux mechanism can be altered [56]. The combination of the herbal and the synthetic remedy can be conjugated using a chemical conjugation as well as a physical combination method. The addition of the lipids will help to cross the BBB as well as act as a P-gp inhibitor [76].

Many techniques, including prodrugs and nanotechnology-based technologies, have been attempted and employed to increase the bioavailability and cellular absorption of RES. The RES is entrapped and transported to the target location by a lipoidal layer and interior hydrophilic layers. Distinct encapsulating methods have varied advantages and disadvantages, as well as different impact qualities [77]. As a result, a nanocarrier system capable of extending molecule life while preserving biological and physical features is required. This study focuses on unique RES management and execution strategies for increased treatment effectiveness [40].

Several techniques for delivering RES to the brain have been researched in recent years. Several materials have been utilized to encapsulate RES and increase its activity. Various drug delivery systems have been reported to have advantageous qualities such as increased stability, bioavailability, and biocompatibility. Several methods of extending the duration that NPs are in circulation may be employed. One of the most common approaches is the attachment or adsorption of many molecules to the surface of the NPs [7]. PEG and polysorbates, which are hydrophilic stabilizers, can be utilized. These molecules also provide steric stability to the NP surface, making it simpler to bind other ligand moieties such as antibodies, aptamers, and proteins recognized by BBB receptors [78]. These ligand molecules can aid the immune system in recognizing and eliminating NPs. PEG molecules on the surfaces of the NPs, on the other hand, prevent this behavior.

Targeted molecules might be added to the surface of the NP to increase the bioavailability of the encapsulated RES [79]. Because these ligand moieties connect to the BBB’s existing receptors, NPs may be actively transported across the barrier, enabling brain tissue targeting. Electrostatic forces contribute to the binding of positively charged ligands to the luminal surface of BBB cells. Because the receptor for the ligand must be overexpressed at the BBB, it must be carefully targeted to brain tissue to prevent negative effects on healthy tissues while increasing RES accumulation. To avoid competitive binding of the natural ligand, the saturation of the receptor must also be carefully regulated [80].

Temozolomide is the standard treatment for glioblastoma, although it fails owing to numerous resistance mechanisms and biological obstacles [81]. The O (6)-Methylguanine DNA-methyltransferase (MGMT) protein is associated with temozolomide resistance because it repairs DNA damage caused by temozolomide-induced methylation. Huang and colleagues observed that RES prevented the activation of the NF-kB transcription factor in glioma cells, which is essential for MGMT activation [82]. As a result, RES enhances temozolomide effectiveness by reversing therapeutic resistance. According to previous research, RES improved the chemosensitization of glioma cells to temozolomide activity by altering many signaling pathways and producing apoptosis and cell cycle arrest [83].

## 9. Possible Route of Administration

Nowadays, most antineoplastic drugs for BT are administered either systemically, intravenously, or orally based on the drug’s absorption. Although certain drugs have features that allow them to enter BTs, the constraints discussed above prevent most chemotherapies from being delivered [84].

The delivery of a drug intra-arterially (IA) is intended to increase the quantity of a drug delivered to a vascular region by bypassing first passage metabolism [85]. Nevertheless, for medications that travel efficiently through the CNS, reside time may be reduced, resulting in reduced effectiveness. So far, IA delivery alone has not resulted in a better prognosis for BT patients. Iatrogenic disruption of the BBB (BBBD) before standard chemotherapy has proven to have better outcomes. In preclinical models, IA chemotherapy combined with BBBD enhances the concentration of drugs in the brain parenchyma [86]. The most typical method is osmotic BBBD using drugs like mannitol, supplemented by IA chemotherapy [87]. However, MRI-guided ultrasound-induced BBBD has recently been studied. This provides for more concentrated BBBD and is well tolerated. However, the results of BBBD and IA chemotherapy have varied among tumor types, and the research has been unclear because of small sample sizes. Yet, there were some encouraging outcomes, especially in cancers that have historically been considered difficult to treat, such as brain stem gliomas. For anticancer medicines that are likely to link to tumor tissue (and so not be promptly effluxed) and for chemosensitive malignancies, including primary CNS lymphoma and germ cell tumors, BBBD and focused chemotherapy techniques show the most promise [88]. The negative effects of BBB disruption and IA drug delivery are often ischemic and are most possibly due to catheterization of the vessel for distribution as well as the irritative effects of the medication. It could also be significant neurotoxicity from therapy. BBBD with IA is mainly confined to a small number of skilled institutions since the operations are invasive, complicated, and accompanied by infrequent but possibly harmful comorbidities [89].

In preclinical models, standard radiation treatment used to treat primary and metastatic BT promotes BBB permeability. This is an especially noteworthy finding considering the shown effectiveness of temozolomide in individuals with freshly diagnosed glioblastoma multiforme (GBM) against the low usage of temozolomide alone for recurrent GBM [90]. Therapies that restore the BBB, on the other hand, may, in turn, reduce the drug’s access to the tumor. Others suggest that normalization of the vasculature increases medication delivery by normalizing pressure gradients. If these theories are correct, a series of treatments that differently break but then repair the BBB may enhance drug transport (across a disrupted BBB) while maintaining drug concentrations within the tumor (by mending the BBB), permitting for optimal medication exposure of the tumor. Since 1996, when the US Food and Drug Administration licensed Gliadel (MGI Pharma, Bloomington, MN) for recurrent high-grade gliomas, patients suffering from malignant gliomas have had access to polymer-based drug delivery. Gliadel is a polyanhydride biodegradable polymer wafer impregnated with BCNU (carmustine) that is inserted into the surgical cavity during tumor debulking and has resulted in a 2-month improvement in outcomes in patients with both diagnosed and recurring malignant gliomas [91]. It is generally tolerated, and side effects such as increasing edema, cerebrospinal fluid leakage, and delayed wound healing are uncommon. This method provides local disease management but is hampered by the limited release of BCNU distant from the resection cavity. It is also limited to individuals with restricted cancer who can endure a complete gross resection. Despite these limitations, Gliadel can be quite successful when utilized as a part of a multimodal strategy [92]. Gliadel in conjunction with systemic medicines, such as temozolomide and O6-benzylguanine, is also being studied. Finally, new research indicates that the highest tolerable dose of BCNU in polymers in patients with recurrent gliomas may be 40 mg, rather than the 7.7 mg presently allowed and utilized in clinical practice. Efficacy trials at a higher dosage may yield even better survival outcomes. Gliadel is being used to treat brain metastases. Patients with single brain metastases from distinct solid tumors received resection, Gliadel wafer insertion, or whole irradiation. There were no local herpes outbreaks in 25 patients, and overall survival was 25% after 2 years. Gliadel appeared to manage local illness, and it was well tolerated [93].

Several alternative devices for local distribution have already been developed after Gliadel’s certification. Phase II studies with paclitaxel employing gel technology (ReGel; Protherics, London, UK) in adults with chronic gliomas are now underway. The gel fits the contour of the resection cavity and gradually releases paclitaxel for 4 to 6 weeks [94]. Placing cisplatin-infused plates inside a tumor is another local delivery approach that has shown early potential. According to preliminary findings, they are well tolerated in individuals with newly diagnosed GBM undergoing radiation treatment. The median survival time was 14 months versus 7 months for the control group. All of these techniques are constrained by the necessity for surgical resection and result in a restricted distribution area; hence, they cannot control the full range of infiltrative BT. Furthermore, the regulatory process for such devices is time-consuming, necessitating a fresh testing and approval process for each combination of delivery method and medicine [95]. Local delivery systems, however, provide a direct, well-tolerated, and effective therapy in certain individuals and may serve as the cornerstone of a successful multimodal strategy. Convection-enhanced delivery (CED) uses catheters implanted into and around a tumor to administer anticancer medicines with hydrostatic pressure. It is an appealing strategy for drugs that are too big to penetrate the BBB or too toxic for systemic delivery. That is a very appealing strategy for conjugated, targeted toxin therapy, antibodies, and sometimes even entire cells [12]. The Phase III Randomized Analysis of Convection-Enhanced Delivery of IL13-PE38QQR to Survival Endpoint (PRECISE) trial, which investigated interleukin-13 conjugated to cintredekin besudotox (PE38QQR), and also the TransMID (transferrin-CRM107) trial, which investigated a modified diphtheria toxin (CRM107) conjugated to transferrin in patients with relapsed malignant gliomas. The median survival in the PRECISE trial was 36.4 weeks (compared with 35.3 weeks with Gliadel). The TransMID trial was recently halted at the interval analysis stage to determine the probability of improved overall survival compared with standard second-line glioma therapies, and final results are still pending [96]. CED delivery has been used to test several other antibody-mediated therapies and immunotherapies. All had tolerable toxicity but highly variable efficacy.

CED has many of the same limitations as polymer-based therapies, such as a constrained distribution area and the need for surgery [97]. Furthermore, because drug delivery is dependent on high infusion rates, there may be a higher risk of neurotoxicity from enhanced intracranial pressure. Innately, more catheters installed throughout heterogeneous tumors should result in more delivery; even so, this may be technically complex, and it has not been demonstrated in clinical practice. The most likely case is that other factors, including the rate of efflux from the CNS, proximity to white matter tracks, and bulk flow patterns, influence the delivery, and hence the efficacy, of the infused agent [98]. Because drug distribution is a major limitation of CED, imaging techniques such as fluorodeoxyglucose–positron emission tomography (FDG-PET), diffusion-weighted imaging (DWI), MRI, and single photo emission computed tomography (SPECT) are progressively used to image agents inside the brain after CED [99].

A blood–CSF (B-CSF) barrier exists in addition to the BBB. The barrier is made up of fenestrated endothelial cells on the luminal side as well as tightly joined epithelial cells without fenestration on the basolateral side (the ependyma). Multidrug resistance transporters are expressed all along the B-CSF barrier, limiting drug entry from systemic circulation to the CSF and clearing drugs attempting to enter the CSF from the brain [100]. Given that leptomeningeal disease affects up to 5% to 15% of patients with solid tumors, there have been concerted attempts to improve drug delivery to CSF spaces. There are three methods for delivering drugs into the CSF spaces: intrathecal, intraventricular, and intracavitary. Multiple processes are needed for this procedure, and drugs may have limited numbers all across CSF pathways [101]. Intraventricular administration of drugs necessitates the use of hardware (i.e., implanted reservoirs), and it is likely to lead to increased drug volume distribution throughout CSF pathways. Even though it is well tolerated, there are a few side effects, such as arachnoiditis, meningitis, and focal neurologic injury. Besides this, if there are CSF flow abnormalities, each of these methods will result in a reduced volume of distribution (and potentially increased toxicity). Because these abnormalities are frequent in people with leptomeningeal disease, CSF flow research must be ordered before starting any CSF drug transfusion [102].

Anticancer therapies can be delivered directly to tumor cysts or cavities via implanted reservoirs. In patients with recurrent malignant glioma, for example, a phase II trial of chlorotoxin combined with the radioisotope 131I (131I-TM-601) infuses radioactive therapy into the tumor resection cavity via an Ommaya reservoir. This method allows for repeat dosing but is limited due to the limited volume of distribution [103].

**Figure 5:**
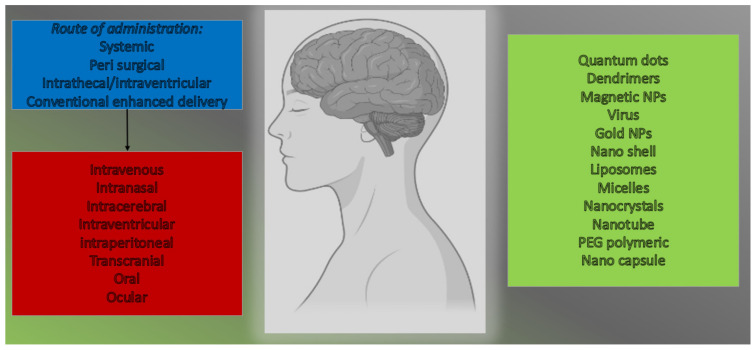
Possible route of administration to target the brain.

The anticancer activity of RES can be influenced by different administration techniques. Bioavailability improves when RES is protected from considerable metabolization in the gastrointestinal tract and liver, which is especially essential in intracranial cancers [104]. Following RES mitigation, there is a wide range of apoptotic foci with reduced Cyclin D1 staining, as well as increased autophagy with increased autophagy-related proteins LC3 as well as Beclin 1, and improved autophagy with increased autophagy-related proteins LC3 and Beclin 1. As a result, novel therapeutic approaches are desperately needed [105]. The breakdown of the BBB is an important step in the development of therapies for central nervous system (CNS) syndromes. In this situation, the intranasal route of drug administration has also been projected as a non-invasive alternate route for straight targeting of the CNS [106]. This method of drug administration avoids the BBB, reducing systemic toxicity. Some formulations, primarily based on the use of nano-sized and nanostructured therapeutic agents, have recently been developed to improve nose-to-brain transport [107]. The purpose of this research is to deliver a summary of the approaches advanced for delivering anticancer compounds via nasal administration to mitigate GBM. Particular attention will be paid to the properties of nanomedicines proposed for nose-to-brain delivery [107]. Preclinical and clinical data suggest that nasal delivery of anticancer drugs could be a game-changer in the fight against GBM. The BBB keeps potential mitigation moieties from entering the brain. Directly targeting the brain via olfactory and trigeminal neural pathways, even while passing through the BBB, has grown in importance for the delivery of a wide range of therapeutics to the brain. The intranasal route of administration delivers drugs to the brain without causing systemic absorption, avoiding side responses, and increasing neurotherapeutic potency [108]. Various drug delivery systems (DDSs) for targeting the brain via the nasal route have been researched in the last several decades. For example, liposomes, nanoparticles (NPs), and polymeric micelles are examples of novel DDSs that can be responsive tools for targeting the brain without causing toxicity in the nasal mucosa and CNS [109]. Mayuri and Shilpa, in 2017, found out that cubosomal in situ nasal gel containing RES was found to be effective for brain targeting [110]. Valentina et al. developed lipid microparticles (LMs) that were untreated or coated with chitosan and contained the neuroprotective polyphenol resveratrol for nasal delivery [111]. The possible route of administration to target the brain is given in Figure 5.

## 10. RES about Autophagy in Brain Cells

Depending on the dosage and other parameters (such as exposure period and cell nutritional condition), resveratrol can induce autophagy in brain cells [112]. However, it is unknown if the mitochondrial effects of resveratrol and autophagy activation are related. Because cells use autophagy as an adaptive strategy during various types of stress, and mitochondrial biogenesis and dynamics also play a role in stress response, it would be interesting to see how autophagy and mitochondrial biogenesis and dynamics interact in these conditions when resveratrol is present [113]. Autophagy is connected to mitochondrial function because it regulates the number of damaged organelles and proteins.

According to this view, autophagy is required for mitochondrial function. Synuclein accumulation in the cytosol and mitochondria may restrict mitochondrial function while enhancing reactive species generation. More research is needed to completely understand the mitochondria–autophagy relationship. This is a difficult issue to research since, for example, the stress response differs depending on the cell type [114]. Furthermore, due to resveratrol bioavailability, in vitro outcomes may differ from in vivo effects. Autophagy starts in reaction to chemical stresses, which may reduce therapeutic effectiveness [115]. This is especially essential when looking for ways to induce apoptosis in cancer cells [116].

## 11. Control of Cellular Proliferation, Apoptosis, and Differentiation

RES may be able to target a range of chemicals involved in the genesis and progression of BTs. Because p53 mutations or abnormalities in its pathway are observed in the majority of glioma tumors, the tumor suppressor cytoplasmic protein p53 might be an antiproliferative target of RES [117]. P53 causes cell cycle arrest during the G1/S phase in response to stress and DNA damage, limiting DNA replication in mutant cells. RES promotes the accumulation and phosphorylation of the p53 protein, which increases its expression [75]. RES also inhibits cell cycle progression by downregulating the enzyme cyclin D1, which is involved in the transition from the G1 to the S phase of the cell cycle [118].

RES also inhibits the ribonucleotide reductase enzyme, which is necessary for DNA synthesis during the S-phase of the cell cycle, resulting in cell cycle arrest. The transcription factor STAT3 is another well-studied RES target. The STAT3 signaling pathway, which is dramatically elevated by phosphorylation in BTs, influences the aggressiveness and recurrence of glioma tumors [119]. Cell cycle progression regulation, mediating the transcription of many different genes, participation in many cellular mechanisms such as apoptosis, angiogenesis, and tumor invasion of adjacent tissues, and acting as an important indicator for the patient’s survival rate, tumor growth, and progression are all part of its role in the carcinogenesis of these tumors [54].

The origin, progression, and recurrence of glioblastoma multiforme are all dependent on glioblastoma-initiating cells. Because glioblastoma-initiating cells are so resistant to temozolomide, eliminating them is seen as a vital step in ensuring the long-term survival of GBM patients [120]. RES, a naturally occurring polyphenol, has sparked interest as a cancer prevention and mitigation agent. It is uncertain if RES can make glioblastoma-initiating cells more sensitive to the chemotherapeutic medication temozolomide. RES was proven in this work using patient-derived glioblastoma-initiating cell lines to sensitize glioblastoma-initiating cells to temozolomide both in vitro and in vivo. Additionally, RES increased temozolomide-induced apoptosis in glioblastoma-initiating cells by activating the DNA double-strand break/pATM/pATR/p53 pathway [117]. Several studies have shown that combining temozolomide with RES mitigation for glioblastoma patients, especially those with a large number of glioblastoma-initiating cells in their cancers and a poor response to temozolomide, might be beneficial [6]. The proapoptotic, antiproliferative, and anti-inflammatory characteristics of RES are regarded as the most important anticancer mechanisms in many types of cancer. Cell proliferation in mammals is separated into two stages: (a) the cell cycle, which includes genetic material duplication and division, and (b) cell growth, which is affected by several growth hormones [121]. G1 (Gap 1), G2 (Gap 2), S (synthesis), and M are the four cell cycle stages that are primarily controlled by cyclin-dependent kinases (CDKs) that function in a complex with their cyclin partners (mitosis).

Apoptosis, also known as programmed cell death, results in cellular death due to cell shrinkage, loss of cell membrane integrity, and DNA breaks. Apoptosis is induced by a variety of intracellular and extracellular stimuli, and the lack of apoptotic responses is required for tumor growth and treatment resistance. RES has been shown in several studies to trigger apoptosis in glioma cells. Tseng et al. (2004) discovered that RES increased apoptosis in glioma cells in animal tests [122]. In U251, U87, and C6 glioma cells, RES can induce apoptosis via caspase-8 or caspase-9-dependent pathways. These caspases both operate as initiators, prompting effector caspase-3 to disintegrate glioma cells into apoptotic bodies (through hydrolytic cleavage). Survivin, a caspase inhibitor, is likewise inhibited by RES, hastening apoptosis. The current study found that RES can activate caspase-3 in glioma cells by blocking the intracellular signaling pathway PI3K/Akt/mTOR [123]. The PI3K/Akt/mTOR pathway is hyperactive in glioma tumors, resulting in decreased apoptosis and quicker tumor progression, and it is frequently associated with drug resistance. Another biological avenue via which RES may cause apoptosis is inhibiting the protein kinase C signaling pathway. Protein Kinase C overexpression has been linked to glioma cell proliferation [124].

Apoptosis, often known as cell death, is the result of an irreversible halt in the cell cycle. By interrupting the cell cycle during the S phase, RES could delay cell cycle progression and limit the development of rat C6 glioma cells [125]. Oncogenic microRNAs (miRs) such as miR-19, miR-21, and miR-30a-5p were shown to be repressed in glioma cells, which was connected to alterations in the expression of their target genes such as p53, NF-KB, EGFR, STAT3, COX-2, and the PI3K/AKT/mTOR signaling pathway.

Furthermore, RES suppressed cancer development and extended life in rats with intracranial C6 glioma. In human GBM cell lines, RES produced S-G2/M cell cycle arrest, which was followed by a rise in pCdc2(Y15), cyclin A, E, and B levels, and a reduction in cyclin D1 [42]. According to a recent study by Laaniste et al., the M2 gene-regulatory network, which consists of 177 genes and guides G2 to M development in low-grade gliomas, is drastically downregulated by RES [126]. Even at nanomolar RES concentrations, the transcription of late cell cycle genes such as FosM1 and B-Myb was most affected. Deregulation of precursor cell differentiation is critical in the formation of BTs. As a result, differentiation-promoting drugs may be able to delay the growth of GBM and medulloblastoma in patients, diminish cancer resistance, and avoid recurrence [99], as evidenced by decreased nesting (a stem cell marker) and increased expression of the glial acidic fibrillary protein (a mature glial cell marker) and beta III-tubulin (a neuronal differentiation marker) in human U87MG cells over time. Numerous investigations have demonstrated that RES has anticancer properties at the level of posttranscriptional gene expression regulation [127]. Thrombotic Thrombocytopenic Purpura (TTP) is an RNA binding protein that binds to AU-rich regions in target mRNAs and promotes the deadenylating and destruction of target transcripts, including proto-oncogenes, antiapoptotic genes, immune regulatory genes, and others. In U87MG human glioma cells, RES increased TTP expression, causing apoptosis and reducing cell growth [128].

## 12. Cellular and Molecular Mechanisms

RES is a phytochemical that belongs to the stilbene family and is pleiotropic. Even though it is only found in tiny amounts in grape products, it has been the focus of several preclinical investigations in cellular and animal models to study its anticancer potential [129]. RES’s molecular mechanisms include extracellular growth factor and receptor tyrosine kinase signaling pathways, multiprotein complex formation and cell metabolism, cell proliferation and genome instability, cytoplasmic tyrosine kinase signaling (cytokine, integrin, and developmental pathways), signal transduction by the transforming growth factor superfamily, apoptosis, immune surveillance, inflammation, and hormone signaling [130]. RES also showed promise in the fight against multidrug resistance; when combined with 5-fluorouracil and cisplatin in adjuvant therapy [131], it displayed additive and/or synergistic responses, enhancing cancer cell chemo sensitization [132]. RES has been discovered as a potential multi-target anticancer mitigation due to its proclivity to act on several targets. Preclinical research indicates that phytochemicals interact with a wide range of molecular and biochemical targets implicated in chemical carcinogenesis, a three-stage process that begins with cancer and progresses through promotion, progression, invasion, and metastasis. In the case of RES, many chemo preventive and chemotherapeutic techniques to halt, prevent, or reverse carcinogenesis stages have been investigated [133]. RES can disrupt a variety of faulty signaling pathways, allowing it to act as a functionally pleiotropic medication, targeting several targets in cancer cells while inflicting minimal harm to healthy cells. Some of the most significant cellular changes are accelerated cell cycle checkpoint transitions with abnormal cell proliferation, genome instability, abnormal response to signals or other stimulators of programmed cell death, uncontrolled neo angiogenesis, increased oxidative stress, overstimulation of growth regulatory hormones, and changes in host immune responses [134]. Anti-inflammatory, antioxidant, and immunomodulatory properties aid in the reduction of oxidative stress-induced damage (protein oxidation, DNA damage, and lipid peroxidation), as well as increasing immunological cancer surveillance. By inhibiting the monooxygenase cytochrome P450 isoenzyme CYP1A1, which is responsible for xenobiotics metabolism in the liver, RES can function as a blocking agent, preventing the transition of a procarcinogen to a carcinogen [135].

## 13. Lipids and Their Advantages in BT

BTs are the tenth highest cause of mortality among all cancer patients globally, and patients continue to face bleak prognoses and few treatment options. This illness has a 14-month median survival duration following diagnosis [136]. There are several factors that make BT therapies ineffective, including the BBB, which limits drug penetration into the brain, and surgical limitations in removing tumors due to their location in the central nervous system (CNS) without affecting survivors’ quality of life [137]. Nanomedicine has the potential to be a game changer in this regard. Furthermore, it has the ability to personalize therapy and overcome the BBB in order to improve anticancer medication administration and distribution [138].

Numerous nanoparticles, including liposomes, polymeric nanoparticles, micelles, dendrimers, and gold nanoparticles, have been studied for their anticancer drug delivery efficacy. Lipids are amphiphilic biomolecules that are normally insoluble in water and have been employed in medicinal formulations such as emulsions, lotions, and ointments [139]. They are nontoxic, biocompatible, and biodegradable, which are the most important criteria for designing nanoparticles for biological purposes. Furthermore, functionalizing these nanoparticles and transforming them into smart delivery systems is a rather simple process [140].

Similarly, biocompatible natural substances and/or a mixture of natural lipids are used to make solid lipid nanoparticles (SLNs) and nanostructured lipid carriers (NLCs). Because of the similarities between these nanoparticles and cell membranes (both chemically and physically), they have minimal effect on the extracellular/intracellular environment and allow a well-regulated release of various encapsulated biologically active substances over lengthy periods of time. Because of their modest average size, they easily move through the circulation, avoiding macrophage absorption [141]. SLNs and NLCs, like other lipid or polymeric nanoparticles, can be modified with a variety of targeting ligands such as aptamers, peptides, antibodies, or even tiny targeting molecules. Because of the extensive tuning process that SLN and NLC went through for delivering nucleic acids, these nanoparticles were regarded as suitable candidates to transport mRNA for COVID-19 vaccines (Zhang). Furthermore, due to their great biocompatibility and minimal immunogenicity, SLNs and NLCs are regarded as the ideal options for addressing CNS disorders [142].

## 14. Formulation-Based Approaches

Despite recent advances in brain glioma detection and therapy, it is argued that there has been no meaningful reduction in BT-related mortality rates due to difficulties in primary stage identification. According to the facts and estimates, further progress is needed [143]. Such advancements include earlier identification and therapeutic regimens that will be more precisely taken up by BT cells while also causing less off-target damage [144].

SLNs are another sophisticated type of nanocarrier that might be used in the targeted delivery of a drug. They are made of biocompatible lipids and are deemed safe. They have a size range of 10–1000 nm and are generated by dispersing lipids into water or an aqueous surfactant solution. They combine the advantages of liposomes and PNPs and exhibit remarkable physiological stability [145]. Furthermore, no harmful organic solvent is required in the synthesis of SLNs, making them safe for usage. They may combine both hydrophilic and hydrophobic chemicals, which is notably beneficial in protein or peptide delivery. They can be viewed as a very adaptable platform for BT imaging and therapy [146]. Several BTs targeting SLNs and their in vitro and in vivo effectiveness have been researched during the last two decades [147]. The findings of these investigations have been found to improve the efficacy of chemotherapeutic drugs while decreasing their negative effects. Polysorbate-coated particles were shown to boost CNS pharmacological effects, but Poloxamer coatings were ineffective. Brain-targeted, Poloxamer stabilized stearic acid camptothecin-loaded SLNs were tested in mice following both oral and intravenous injection. The concentration maximum (C_max_) rose by 180% after IV administration of SLNs. The area under the curve (AUC)/dose and Mean residence time (MRT) of SLNs were raised by 10.4 and 4-fold, respectively. Jain et al. recently created transferrin (Tf)-conjugated SLNs (Tf-SLNs) and tested them for temozolomide (TMZ) transport to the brain for GBM treatment. The intensity of fluorescence seen in cellular uptake studies was higher in Tf receptor-targeted SLNs than in non-targeted SLNs [148]. Martins et al. investigated the capacity of camptothecin-loaded SLNs to enter the brain parenchyma after passing through the BBB in another investigation. They developed camptothecin-loaded SLNs for brain targeting and demonstrated that SLNs had a better effect on brain targeting than non-encapsulated drugs [149].

SLNs comprise one or more solid lipids and bioactive chemicals entrapped inside the lipid matrix and stabilized by a surfactant or polymer. They are solid at both room and body temperatures and have typical sizes of 50 to 100 nm. SLNs are appealing for drug delivery systems because they are made from biocompatible and biodegradable lipids, making them safe and viable for large-scale manufacture at a low cost [150]. SLNs have superior physical stability and increased hydrophobic drug bioavailability. Furthermore, they preserve the integrated medication from degradation and allow for regulated drug release (sustained or rapid), while their manufacture does not necessitate the use of harmful organic solvents. Furthermore, by grafting certain surfaces with antibody receptors, proteins, and peptides, they may actively target the infected site. Purified triglycerides (e.g., tripalmitin, tristearin), complex glyceride mixes (e.g., Witepsol^®^ W35), and waxes (e.g., Carnauba wax, Cetyl palmitate, and Beeswax) are utilized in the production process. Bioactive molecules are inserted into SLNs using one of three fundamental models: drug-enriched shell, drug-enriched core, and homogeneous matrix. SLNs transition between a form (α: less ordered polymorph with hexagonal chain packing) and form β′ (β′: orthorhombic perpendicular chain packing) to a β form (β: triclinic parallel chain packing). This shift to a more ordered form phase is responsible for decreasing loading capacity and increasing drug ejection (α < β′ < β). As a result, the existence of a high degree of order lowers those flaws in the crystal lattice, resulting in drug ejection and a decrease in loading capacity. A less ordered solid lipid matrix, on the other hand, is required to have a larger loading capacity for the integrated medicines [151]. As a consequence, the lipid matrix, which formerly consisted of comparable molecules such as tristearin, is replaced with more complex lipids (mono, di-, triglycerides with varying chain lengths) to improve loading capacity and limit drug ejection by increasing defects. Aside from drug ejection and restricted loading capacity, SLNs suffer from high dispersion water content (more than 70%). As a result, nanostructured lipid carriers (NLCs) have been created to address the shortcomings of SLNs [152].

**Figure 6:**
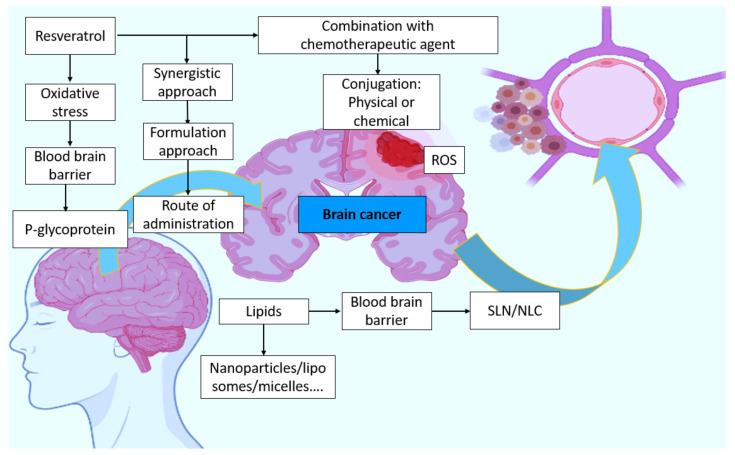
Hypothetical concept of resveratrol involvement in brain cancer and possible strategies.

NLCs are the second generation of SLNs and are created by combining liquid and solid lipids. Castor oil, oleic acid, palmitic acid, olive oil, linoleic acid, and coconut oil are the most often utilized liquid lipids. The liquid lipid to solid lipid ratio ranges from 30:70% to 0.10:99.9%, and the particles are stabilized by adding 0.5–5% surfactant solutions. Even though NLCs contain liquid lipids, they remain solid at room and body temperatures. Many of the restrictions associated with SLNs can be overcome with the development of these nanosized compartments. NLCs boost drug loading capacity, manage release qualities, improve encapsulation efficiency, and extend chemical stability in this respect [153]. Furthermore, as compared to SLNs, adding liquid lipids to the lipid matrix inhibits drug ejection and thereby boosts drug incorporation capability. Furthermore, additional flaws in the crystal structure are generated to create more room for drug inclusion. NLCs are suitable for a variety of delivery methods, including ocular, topical, oral, and parenteral. The loading capacity rises, and the integrated medicine is not ejected from the core formulation. Type I, or imprecise type, is distinguished by crystal formation defects in which a portion of the solid lipid is replaced by liquid lipids to increase the ratio of imperfections in the lipid matrix’s lattice, resulting in greater free space for drug accommodation. As a result, the loading capacity rises, and the integrated medicine is not ejected from the core formulation [147]. In Type II, or amorphous, the medication is incorporated in a solid amorphous (formless) matrix in this type. Instead of the imperfect crystal, liquid lipids such as hydroxyoctacosanyl hydroxy stearate and isopropyl myristate are combined to produce -a polymorph (less ordered polymorph). Amorphous type NLCs are often preferred over defective NLCs because they are more stable and have a larger loading capacity. Multiple oil-in-fat-in-water (O/F/W), or type III, is made up of an oil-in-fat-in-water dispersion and is developed from water-in-oil-in-water (w/o/w) emulsion. This is often created by heat homogenizing solid lipids with high volumes of liquid lipids. After cooling, oily nano-compartments form as a result of phase separation of the surplus liquid lipid. This form of NLC is very appealing, especially for pharmaceuticals that are more soluble in liquid lipids than in solid lipids, and it has a large loading capacity [154]. Furthermore, the exterior lipid matrix surrounding the nano-compartments acts as a barrier, preventing drug leakage and allowing for regulated release [139]. A hypothetical concept is given in Figure 6.

## 15. Conclusions

Headache, which is common among the population, is an unavoidable symptom of brain cancer. When it goes unnoticed or undiagnosed, it may lead to the development of cancer or its invasion of other organs. Rapidly growing brain cancer causes oxygen deprivation and hypoxia. This causes apoptosis and increases the stimulation of reactive oxygen species, both of which are harmful to non-cancerous cells. Antioxidants are compounds that can reduce oxidative stress. RES is a low-toxicity molecule that targets multiple molecular signal pathways to affect several carcinogenesis-related genes. RES has been shown to have significant anti-cancer potential when used alone or in combination with other anti-cancer drugs and targeted therapies. RES affects brain structure because it crosses the blood–brain barrier. Cell death mechanisms are activated to inhibit cell proliferation while also reducing oxidative stress and inflammation to prevent brain cancer. More study is required to address its pharmacokinetic constraints, such as limited human bioavailability, characterize its efficacy in various kinds of BT cells, and identify its precise anticancer modes of action. The development of RES analogs targeted to specific anticancer pathways, as well as greater stability and bioavailability in organisms, could result in improved anticancer efficacy and therapeutic usage. This can be accomplished by altering gene transcription, which affects metabolism and other critical signaling pathways. Cell death, immune response, and protein processing are a few examples. As a result, determining how cancer cells respond to changes in their microenvironment, such as oxygen levels, nutrient availability, or mitigation-induced damage, is difficult. To the best of our knowledge, no human clinical trials assessing the responsiveness of RES against brain cancers have ever been conducted. According to the current study, RES administration is useful in the treatment of brain disease and also in brain cancer by reducing viability, proliferation, and migration via molecular pathway modification.

## Data Availability

Not applicable.
